# Mechanistic Models of Cellular Signaling, Cytokine Crosstalk, and Cell-Cell Communication in Immunology

**DOI:** 10.3389/fimmu.2019.02268

**Published:** 2019-09-25

**Authors:** Martin Meier-Schellersheim, Rajat Varma, Bastian R. Angermann

**Affiliations:** ^1^Laboratory of Immune System Biology, National Institute of Allergy and Infectious Diseases (NIAID), National Institutes of Health (NIH), Bethesda, MD, United States; ^2^Xencor Inc., Monrovia, CA, United States; ^3^Translational Science and Experimental Medicine, Early Respiratory, Inflammation and Autoimmunity, BioPharmaceuticals, AstraZeneca, Gothenburg, Sweden

**Keywords:** computational models, cellular signaling, cytokine crosstalk, multi-scale modeling, rule-based modeling

## Abstract

The cells of the immune system respond to a great variety of different signals that frequently reach them simultaneously. Computational models of signaling pathways and cellular behavior can help us explore the biochemical mechanisms at play during such responses, in particular when those models aim at incorporating molecular details of intracellular reaction networks. Such detailed models can encompass hypotheses about the interactions among molecular binding domains and how these interactions are modulated by, for instance, post-translational modifications, or steric constraints in multi-molecular complexes. In this way, the models become formal representations of mechanistic immunological hypotheses that can be tested through quantitative simulations. Due to the large number of parameters (molecular abundances, association-, dissociation-, and enzymatic transformation rates) the goal of simulating the models can, however, in many cases no longer be the fitting of particular parameter values. Rather, the simulations perform sweeps through parameter space to test whether a model can account for certain experimentally observed features when allowing the parameter values to vary within experimentally determined or physiologically reasonable ranges. We illustrate how this approach can be used to explore possible mechanisms of immunological pathway crosstalk. Probing the input-output behavior of mechanistic pathway models through systematic simulated variations of receptor stimuli will soon allow us to derive cell population behavior from single-cell models, thereby bridging a scale gap that currently still is frequently addressed through heuristic phenomenological multi-scale models.

## Introduction

Immune cells have been found to play important roles for processes ranging from embryogenesis to tumor clearance to host defense against pathogens (1). What allows them to perform such diverse tasks is the ability to respond to a great variety of different signals, many of which reach them simultaneously (2, 3), and adjust their behavior through communication with other, immune and non-immune, cells (4–6). When their response mechanisms fail to induce the appropriate action, clearance of pathogens or tumor rejection may fail and immune-pathologies such as autoimmune, or inflammatory diseases may develop.

In our efforts to understand immune cell function, the challenge of understanding multi-signal cellular responses or multi-cellular communication and how these integrate at the tissue level is perhaps the most daunting since it seems to go directly against the paradigm of reductionism that has brought forth most of the insights science, not just immunology, is based upon. Indeed, approaching this challenge requires more comprehensive data than classical one-condition-one-readout assays. In model organisms, such as mice, lack of approaches to generate data elucidating cellular behavior under various conditions is no longer the main problem, though. Highly multiplexed assays can be employed and allow us to glimpse into cellular protein expression levels including post-translational modifications (7) and genomic states, increasingly also at the single-cell level (8, 9). Multi-parameter *in-vivo* microscopy shows us where cells are, where they go and with whom they interact (10–12). However, such data are dots waiting to be connected into mechanistic hypotheses: Even though we may be able to use the data directly to predict disease progression probabilities through artificial intelligence based informatic approaches we need to understand mechanisms to devise therapeutic interventions. Moreover, the invasiveness of many assays prevents us from generating similar data in humans, both in clinical practice or in a research setting. Thus, we are facing the conundrum that we are able to generate highly detailed data but cannot be certain which of the predictions we derived from the data will translate to humans.

Here, we will discuss how the complexity of some of these challenges may be addressed using *mechanistic* computational models using an approach that can clearly state even complex biological hypotheses involving multiple overlapping signals and, sometimes, may permit to test them directly through simulations. We will first explain how such models can be concise and flexible representations of knowledge and hypotheses and, along the way, demonstrate that these representations can be fully accessible to researchers without modeling experience. Then, we will use the modeling approaches we introduced to investigate multi-parametric manipulations of a simple example pathway (a simple model of G-protein coupled signaling and its “pharmacological” manipulation) before illustrating how computational models can be used to explore possible mechanisms of crosstalk in immune signaling pathways. Finally, we will briefly discuss how to extrapolate from single-cell models to models of communicating cell-populations that could serve, for instance, as a basis for more realistic pharmacokinetics-pharmacodynamics (PK-PD) simulations (13) to improve practical applications of basic immunological research.

## Modeling Signaling Pathways Based on Molecular Interactions

Cellular responses toward stimuli they receive emerge from interactions among proteins, lipids, and sugars mediated through specific binding sites. Sequences of such interactions are frequently depicted as networks or pathway diagrams linking, for instance, phosphorylation of a given protein domain to the recruitment of other proteins that subsequently induce or undergo further biochemical modifications. Mathematical and computational models translate such scenarios into quantitative predictions by describing how the abundances of the involved molecules or their post-translational modifications change over time as a result of the interactions among the network's molecules. Depending on the modeling approach, those predictions are generated by solving differential equations or other, sometimes stochastic, algorithms. Many excellent reviews have been written on computational modeling of cellular behavior. See, for instance (14, 15), or (3, 16) for a focus on mathematical modeling approaches in immunology. Ideally, whatever the approach, the mathematical descriptions should not contain more assumptions than the underlying biological hypotheses. One way to achieve this is, perhaps counter-intuitively, to try to model directly the components and interactions within those biological hypotheses, rather than use abstract elements that are introduced for simplification. This avoids introducing properties that do not follow directly from the modeled biology and that may be difficult to spot for non-modelers, in particular when they are formulated in mathematical terms. Moreover, given that cell-biological, immunological, and biochemical research has assembled a wealth of mechanistic insights, it would be unwise not to take as much as possible advantage of prior knowledge about the constituents and interactions that shape signaling pathway behavior. Finally, building models that incorporate details considered important by experimental biologists allows us to convert model behavior directly into experimental assays for validation since model components have real biological counterparts.

Yet, we are typically lacking many of the parameters required for detailed models, such as protein abundances or kinetic interaction rates, and more experimental data than are usually collected would be needed to determine the values of the unknown parameters through fitting (17). This problem is frequently taken as a motivation to resort to models that incorporate pathway structure but not kinetics [for instance in Boolean models (18)] or abandon prior pathway knowledge altogether in favor of extracting only as much information as the data being modeled provide directly (19). Both such approaches have their merits given the problem of “over-fitting” in models with large numbers of parameters. But we wish to argue that we can use detailed models in spite of parameter uncertainty simply by asking what kinds of behaviors the models can have when taking into account the possible range of their parameters. Exploring crosstalk among cytokine signaling pathways in T cells, we will show that, in contrast to what many theorists would assume, pathway models based on the description of molecular binding sites can have surprisingly little flexibility in their behavior. Thus, the frequently cited *von Neumann* quote about the four parameters that can fit an elephant and five that can make him wiggle his trunk does not always apply.

Another potential hurdle when creating detailed, mechanistic models is that they can be rather large and assembling or maintaining them (i.e., adapting them to new hypotheses) can be laborious and error prone. However, the translation of a pathway diagram (which is, in a way, a model) into a formal language can be done automatically nowadays and in a manner that does not modify the biological content. A number of tools have been developed that can perform such automated translations into computer simulations (see, for instance, http://sbml.org/SBML_Software_Guide). Among them, “rule-based” approaches permit specifying details such as the binding sites that mediate the molecular interactions (20–22), thereby incorporating aspects that can, for instance, help identify molecular binding motives as potential targets for pharmacological modulation through small molecule inhibitors. Finally, constructing models step-by-step by specifying the interactions among its components and then letting algorithms assemble the computational representations of the resulting networks will allow us to consider models that would be too complex for manual construction because of the number of components or because they span several scales (23) or utilize as experimental input very large data sets, for instance based on proteomic studies (24).

## A Simple Example: Modulating a Model of G-Protein Coupled Receptor Signaling

**Figure 1:**
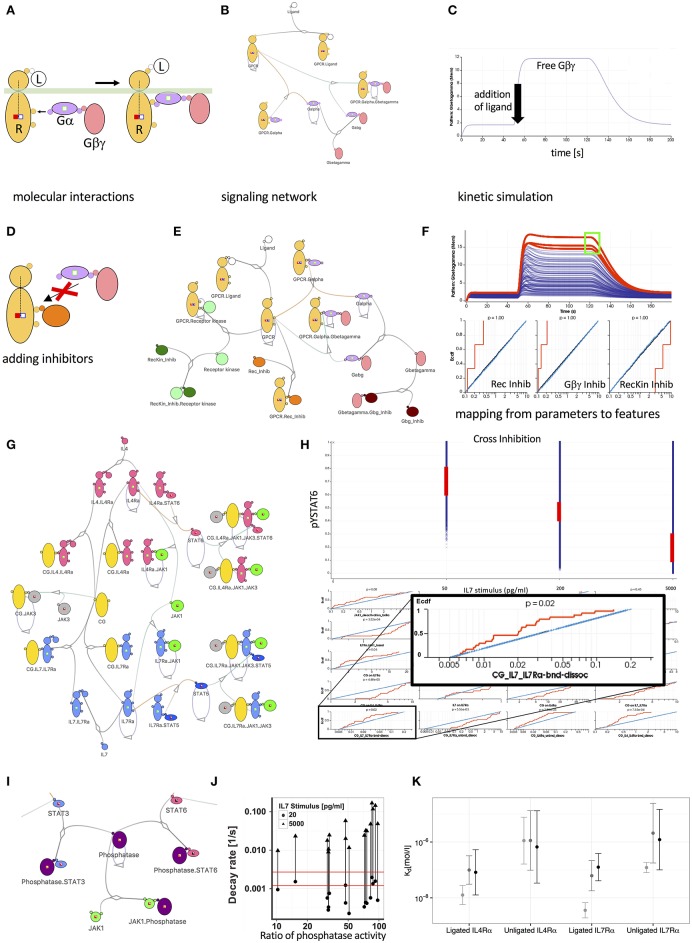
Mechanistic models promote insight into the behavior of signal transduction networks. **(A)** Visualization of G-protein (Gα and Gβγ) recruitment and activation by a ligand bound (L) receptor (R) using the Simmune iconographic notation. Colored boxes indicate required states of the interacting molecules, such as the activation of the receptor (filled red square) or the absence of receptor phosphorylation (empty blue square). Three reaction steps are shown: (i) Gα*βγ* with Gα in the (inactive) GDP state is recruited to the active receptor. (ii) Gα switches from GDP to GTP (green square on Gα becomes filled). (iii) The activated G proteins are released from the receptor. **(B)** Network diagram of a simple GPCR signaling network. Lines connecting different molecules represent possible association or dissociation events. Loops indicate possible state changes, such as the auto-GTPase activity of Gα. Partially filled boxes indicate the presence of states in the model without specifying their values. **(C)** Simulated response of the signaling network shown in **(B)** to exposure with the Ligand. The initial increase of the free Gβγ concentration is due to the model equilibration to a homeostatic state. After 50 s the ligand is added to the model and concentration of Gβγ increases as the G-protein rapidly dissociates. After 120 s a virtual wash of the cell is performed, removing the ligand from the simulation. This leads to a recombination of the G protein subunits and thus a reduction of the concentration free Gβγ. **(D)** Iconographic representation of an inhibitor competing with the recruitment of the G-protein complex to the receptor. **(E)** Expanded network model including receptor phosphorylation by a kinase and inhibitor molecules interfering with receptor-kinase interaction (dark green molecule), formation of the heterotrimeric G-protein (dark brown molecule) and recruitment of the G-protein to the receptor (orange molecule). **(F)** 500 simulated responses of the model in **(E)** to varying inhibitor concentrations. Red lines indicate simulations matching the selection of high Gβγ concentration (green square in upper panel). Empirical cumulative distributions function (Ecdf) of simulation parameters for selected simulations (red), unselected simulations (blue), and total distribution (black). The Ecdf curves are automatically constructed based on the selected curves. The red Ecdf curve increases whenever a parameter value (x-axis) is part of a parameter set that contributes to the selected curves in the upper panel. **(G)** Network representation of a JAK-STAT signaling network downstream of the IL-4 and IL-7 receptors (IL4Rα and IL7Rα) sharing the common gamma-chain. **(H)** Simulated behavior of STAT6 phosphorylation of the model in **(I)** following different doses of IL-7 pre-treatment. Red lines show experimentally observed values and their corresponding parameter distributions in the matching simulations. The inset focuses on the parameter determining the rate of dissociation of the common gamma (CG) chain from the IL7-bound IL7 receptor. The selected phospho-STAT6 levels (red ranges in upper panel) impose clear constraints, ruling out parameter sets with high off rates for the binding between CG and cytokine-bound IL7Rα. **(I)** Expanding the model in **(G)** by a JAK1 induced phosphatase acting on both STAT3 and STAT6. **(J)** The hypothesis of a signal induced phosphatase is inconsistent with experiments, which observed a signal independent decay of STAT6 phosphorylation (indicated by the range between the red lines). In contrast, the simulations predicted at least 10-fold induction of phosphatase activity, as indicated by the lines connecting low and high IL7 stimulus for pairs of simulations that match all other experimental constraints. **(K)** Predicted dissociation constants for the private receptor chains with the γ-chain in the affinity conversion (light gray) and the ruled-out phosphatase induction models (medium gray and black).

Cells use G-protein coupled receptors (GPCR) for a wide range of extracellular stimuli, among them such that guide immune cells to and within lymphoid structures (25). While the GPCR themselves have been a frequent target of potential pharmacological manipulation based on molecular structural studies (26) the downstream signaling events present many not fully explored opportunities for modulation (27). GPCR mediated signaling follows a simple common principle (28): A ligand binds to the receptor's extracellular binding site, thereby inducing changes in the accessibility or affinity of intracellular binding sites that can recruit heterotrimeric G-proteins. In complex with the receptor, the α subunit (Gα) of the G-proteins will more readily exchange a GDP (Guanine-diphosphate) group for a GTP (Guanine-triphosphate) group, and as a result, will lose its high affinity for the Gβγ subunit that subsequently will be released and can activate downstream signaling proteins such as, for instance, Ras. Avoiding the need to write equations or computer scripts, we can use an iconographic representation (see Figure 1A) to represent the sequence of reactions in a “formal” way just as precisely as differential equations would. In fact, the diagram contains additional information about the interacting binding sites. The modeling software Simmune (22, 29) and a recent extension to the Virtual Cell platform (30) permit using such graphical symbols to specify molecular interactions and illustrates how these interactions are linked in the resulting signaling network. These approaches expand the network beyond the manually specified complexes by determining which complexes can form based on the user-specified bi-molecular interactions. Then, they generate computational representations that can be explored through computer simulations and display time courses for the concentrations of these complexes.

### Simulating and Modulating the GPCR Pathway

Once we have a computational representation of a signaling pathway we can not just simulate the kinetics of the concentrations of the involved molecular complexes and, typically, their post-translational modifications. We can systematically vary the parameters in the model to analyze their influence on the behavior of the modeled system. We might, for instance, ask how the affinity of the G-proteins for the activated receptor or the rate at which Gα switches back to its GDP state (the auto-GTPase activity of Gα) shape the characteristics of the response. Experimenting with these rates in the computational model is far easier than altering molecular properties experimentally in the lab.

The possibility to vary reaction rates and molecular concentrations easily in a computational model becomes particularly interesting when starting with a well-established model, such as the GPCR model here, and adding signaling components to identify which combinations of such additions can be used to achieve a desired type of response—a recurring question for pharmacological research on “small molecule inhibitors.” In this example, we added a receptor kinase that phosphorylates the activated receptor and an inhibitor that can associate with the receptor binding site used by the kinase (“RecKin_Inhib”). Furthermore, we added a molecule (“Gbg_Inhib”) that competes with Gα for binding to Gβγ (and thus interferes with the reassembly of the activatable heterotrimeric G protein complex) and a molecule that competes with Gα for binding to the receptor (“Rec_Inhib,” Figure 1D). Varying the concentrations of the three inhibitors, the single response curve shown in Figure 1C turns into a series of time courses (Figure 1F) for the concentration of free Gβγ, each corresponding to a particular set of inhibitor concentrations. Now, we can analyze which features of the curves are compatible with which ranges for the inhibitor concentration parameters. In the diagram, we selected a region (green square) that corresponds to a strong sustained generation of free Gβγ and find that the inhibitors interfering with the association of Gα and Gβγ need to have a low concentration to allow for efficient activation of the G proteins. On the other hand, the concentration of the inhibitor interfering with the kinase phosphorylating the receptor must be high since phosphorylation deactivates the receptor. In this sense, the inhibitor of the receptor kinase actually strengthens the output (see the figure legend for more details). Whereas, these results are simply what we would have predicted intuitively, they illustrate how features can constrain parameter ranges and how we can map between the two.

## Computationally Exploring Cytokine Crosstalk in T Cells

In the previous section, we showed how the features of a simulated model can constrain the ranges of its parameters. In this section, we take advantage of the parameter mapping technique to show that we can identify the limits of what the pathway can do by varying model parameters over a broad range of physiologically plausible values. Exploring these possibilities becomes useful when we want to test whether a model can explain experimentally observed features even if we have only rough estimates for many of its biochemical parameters.

We have recently used this approach (31) to study elements of the common gamma cytokine signaling pathways that are highly important for many aspects of lymphocyte activation, differentiation and survival (32). The cytokine receptors in these pathways all require the common gamma chain (GC) to initiate downstream signals after binding to their specific cytokines, hence the name “common.” The fact that GC is shared among multiple receptor systems means that, depending on the amount of GC and the combined number of receptors that can interact with it, the behavior of the downstream signaling pathways that lead to activation of STATs may be affected when several cytokine signals have to be processed simultaneously by the responding cell. Indeed, stimulating CD4 T cells with the common gamma cytokine IL-7 reduced their responsiveness toward IL-4 and IL-21, two other CG dependent cytokines. Experimental determination of cell surface receptor abundances revealed a limited abundance of CG relative to other private receptor chains. Intuitive first explanations for this cross-suppression would thus posit that the limited abundance of CG leads to competition for this rate limiting signaling component. Paradoxically, however, the observed cross suppression was asymmetric as neither IL-4 or IL-21 were able to suppress IL-7 signaling. Further, only a few ligated IL-7 receptors were required to cause suppression of IL-4 signaling leading us to question whether CG was truly limiting. To explore this quantitative riddle and determine whether CG sequestration can, nevertheless explain the crosstalk, we simulated the model with private IL-4 and IL-7 receptor chains and a shared common gamma chain as well as receptor-associated JAKs and STAT6 and STAT5 as downstream targets of IL-4 and IL-7 signaling, respectively (Figure 1G). Mapping back from the various experimentally observed suppression strengths for different IL-7 doses we found that the private IL-7 receptor chain needs to have an order of magnitude higher affinity for CG than the private IL-4 chain (Figure 1K). Importantly, we found that the IL-7 private chain was required to have a high affinity for CG even before cytokine stimulation (Figure 1K light gray bar for unligated IL7Rα), a result which we confirmed experimentally. Previous hypotheses alternatively suggested that CG is associated with private receptor chains prior to cytokine binding (33, 34) or assumed that private receptor chains gain high affinity for CG only subsequent to cytokine binding (35, 36). Our explorations suggested that both are probably true: CG has a substantial pre-association with some private receptor chains that is further increased upon cytokine binding (Figure 1K light gray bar for ligated IL7Rα).

### Competing Computational Models of CG Pathway Crosstalk

Being able to modify models with less effort than would be required when writing equations or scripts by hand, we computationally explored other mechanisms that could potentially explain IL-7 induced cross suppression. In particular, we explored the hypothesis whether IL-7 induced phosphatases acting on the JAKs at the receptor level or on the STATs further downstream would be compatible with the experimental data on cytokine induced responses and IL7 mediated suppression. Figure 1I shows the model modification that includes such a phosphatase acting on the STATs. Assessing phosphatase activity prior to and after IL-7 stimulation in quantitative experimental assays, we found a much narrower range of activities than would be required for the degree of suppressive crosstalk we had observed (see Figure 1J). In summary, combining multi-dose stimulation data with a detailed model and mapping back from simulations that reproduced the data to parameter ranges we identified quantitative relationships between receptor-ligand affinities and were able to reject alternative models that relied on IL-7-induced phosphatases.

## From Pathways to Cellular Behavior and Cell Populations

**Figure 2:**
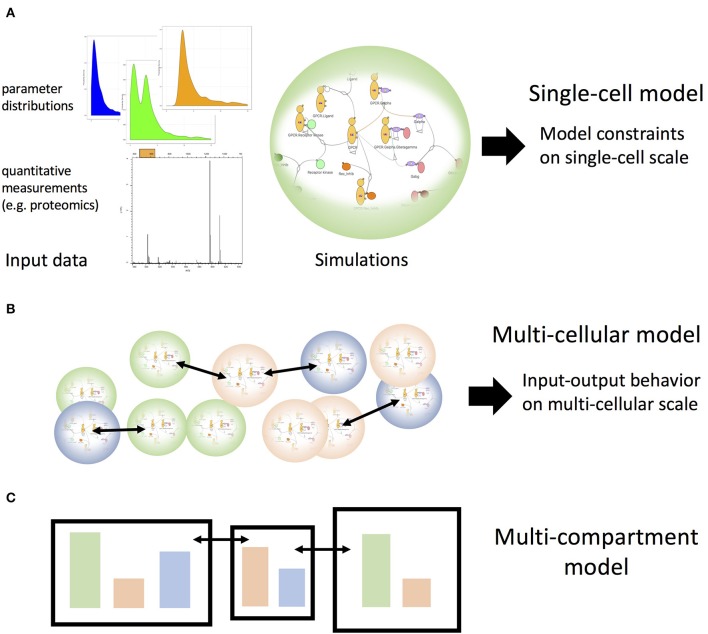
Single-cell models as building blocks for multi-cellular and multi-compartmental higher scale models. **(A)** Experimental data inform mechanistic models of cellular signaling pathways. Parameter scans, such as described in Figure 1 can identify the possible modes of behavior of the single cell models, which subsequently can be used to build systems of interacting coarse-grained cell models to build the scale of interacting cell populations, as illustrated in **(B)**. Iterating this step by extracting the possible patterns of behavior for those cell population models one can build multi-compartment models **(C)** that encompass multiple cell populations and interactions among compartments.

Here, we discussed two strategies for using parameter scans: (i) to explore how model features depend on parameters such as molecule concentrations (how can a model be compatible with the data?) and (ii) to test whether a model can reproduce data at all when allowing the model parameters to vary over a physiologically plausible range (is the model compatible at all with the data?). Both strategies can be used to calibrate or select models at the single cell level (Figure 2A). Building on such calibrated models we can sample the input-output behavior of the single-cell models for such combinations of inputs and cellular states (e.g., abundance of cytokine receptors) that would occur in a multi-cellular system with cells that exchange signaling molecules such as cytokines (Figure 2B). Such a strategy has recently been explored (37). These input-output patterns could then be considered as a collection of simplified models themselves and thus could be employed on the multi-cellular scale to allow for large numbers of cells within a single simulation. Importantly, however, these simplified models would be based on systematic explorations of mechanistic detailed models as opposed to having been designed as simplified models from the beginning. If a combination of stimuli is outside of the range of the previously performed detailed single cell simulations, the collection simplified models could be extended automatically. Using a similar sampling strategy, we will soon be able to extrapolate toward simulations that can generate realistic behavior of compartments comprising many cells of different types while exchanging cells and or molecular messengers (Figure 2C), as is the case for the lymphatic system. This kind of stepwise coarse-graining will be required to link behavior of cell populations to the scale of single-cellular mechanistic models that not only incorporate the current state of biological knowledge but also will allow us to link pathway modulation (e.g., through small molecule inhibitors) to cell population or even tissue behavior.

## Author Contributions

BA developed the Simmune Analyzer discussed in this manuscript. RV planned and supervised the cytokine crosstalk experiments. MM-S and BA developed and simulated the computational models. All authors contributed to writing the manuscript.

### Conflict of Interest

BA is an employee of AstraZeneca. RV is an employee of Xencor. The remaining author declares that the research was conducted in the absence of any commercial or financial relationships that could be construed as a potential conflict of interest.
